# Surgical Management of a Lateral Compression (LC)III Pelvic Ring Fracture With Type I Crescent Fracture-Dislocation: A Case Report

**DOI:** 10.7759/cureus.111097

**Published:** 2026-06-18

**Authors:** João P Leite-Moreira, João Carlos Seixas, João Boavida, António Pais Lopes

**Affiliations:** 1 Department of Orthopaedics and Traumatology, Unidade Local de Saúde de Coimbra, Coimbra, PRT

**Keywords:** crescent fracture-dislocation, iliosacral screw fixation, lateral compression, modified stoppa approach, pelvic ring fracture, polytrauma, preoperative planning

## Abstract

Lateral Compression (LC) pelvic ring fractures represent the most common pattern of pelvic ring injury in the Young-Burgess classification. The Crescent fracture is an uncommon subtype characterized by an iliac wing fracture that disrupts the posterior sacroiliac (SI) complex, creating a crescent-shaped fragment that is inherently rotationally unstable and associated with long-term complications including chronic pain and sexual dysfunction. LC fractures result from a laterally directed force and are subclassified (I-III) based on the degree of posterior ring disruption. We present the case of a 42-year-old male polytrauma patient treated at a Level I trauma center, who sustained an LCIII pelvic ring fracture following a 5-meter fall, comprising comminuted bilateral anterior ring fractures and a posterior ring disruption with a left type I crescent fracture involving the SI joint, with impaction of the mobile iliac fragment into the sacral ala, and a right sacral ala fracture (Dennis I).

No neurological, genitourinary, or additional injuries beyond those described were identified at admission. Preoperative planning included computed tomography (CT) with three-dimensional (3D) reconstruction and a synthetic sawbone pelvic model for implant simulation and plate pre-contouring. Definitive surgical treatment on post-trauma day 10 used a modified Stoppa approach combined with a left iliac window, with de-impaction of the sacral ala and external rotation of the crescent fragment prior to sequential fixation with anterior reconstruction plating, an LCII corridor screw, and bilateral iliosacral screws. At eight-week follow-up, the patient reported no pain, no functional limitation, and no sexual dysfunction, achieving a Majeed Pelvic Score of 96/100, consistent with an excellent functional outcome. This case highlights the critical role of meticulous preoperative planning, multidisciplinary teamwork, and management at a dedicated pelvic trauma center in achieving optimal outcomes in complex LCIII crescent pelvic fractures.

## Introduction

Pelvic ring fractures are high-energy injuries occurring in approximately 3% of all skeletal trauma, frequently associated with life-threatening hemorrhage and multisystem injury [1]. Up to 80% of patients sustain concurrent thoracoabdominal injuries, while genitourinary and neurological complications are reported in 1.6-25% and 18-64% of cases, respectively [2,3]. Hemodynamic instability from uncontrolled hemorrhage remains the leading cause of early mortality [1,2]. Given their complexity and the risk of systemic complications, optimal management of these injuries requires a multidisciplinary team within a Level I trauma center with dedicated expertise in pelvic trauma.

The fracture pattern correlates directly with the direction of the applied kinetic energy. According to the Young-Burgess classification, Lateral Compression (LC) injuries are the most common pelvic fracture pattern, representing 60-70% of cases, followed by Anterior-Posterior Compression (APC, 15-20%) and Vertical Shear (VS, 5-15%) [4]. LC fractures result from a laterally directed force and are subclassified (I-III) based on the degree of posterior ring disruption. LCIII injuries represent the most severe variant, in which the internal rotation force applied to the ipsilateral hemipelvis leads to contralateral anterior sacroiliac ligament disruption with external rotation of the contralateral hemipelvis.

The Crescent fracture is an uncommon subtype of LC injury first described by Day et al. [5], in which the fracture line traverses the posterior ilium, creating a crescent-shaped fragment that remains attached to the sacrum via the intact posterior SI ligaments. These injuries are inherently rotationally unstable and, if inadequately treated, are associated with long-term complications including chronic pain and sexual dysfunction [5]. Depending on the proportion of the SI joint that is disrupted, they are classified into three types: type I (large crescent fragment, with less than one-third of the SI joint disrupted), type II (intermediate-size crescent fragment, with between one- and two-thirds of the SI joint disrupted), and type III (small crescent fragment, with more than two-thirds of the SI joint disrupted). Correct classification is essential, as fragment size determines whether anterior fixation alone suffices or a combined anterior-posterior approach is required. We report a case of an LCIII pelvic ring fracture with a type I crescent fracture-dislocation managed at a Level I trauma center, with preoperative synthetic sawbone model simulation and a sequential fixation strategy, achieving excellent functional results at eight weeks.

## Case presentation

A 42-year-old male electrician presented to the Emergency Department of a Level I trauma center following a fall from a height of 5 meters, sustaining blunt trauma to the head and left hemibody. On primary survey, the patient was hemodynamically stable, with no neurological deficits and no genitourinary injuries. A Focused Assessment with Sonography for Trauma (FAST) examination excluded intra-abdominal hemorrhage. Computed tomography (CT) of the head ruled out intracranial injury. Targeted plain radiographs (Figure 1) and subsequent CT of the pelvis with three-dimensional (3D) reconstruction (Figures 2, 3A-3B) identified displaced and comminuted fractures of the bilateral iliopubic and ischiopubic rami involving the anterior ring, a displaced and comminuted left crescent fracture of the iliac wing with involvement of the SI joint (type I) with impaction of the mobile iliac fragment into the sacral ala, and an aligned right sacral ala fracture (Dennis zone I). Three left-sided rib fractures were also identified. No additional injuries were identified at admission. The overall injury pattern was classified as an LCIII pelvic ring fracture.

**Figure 1:**
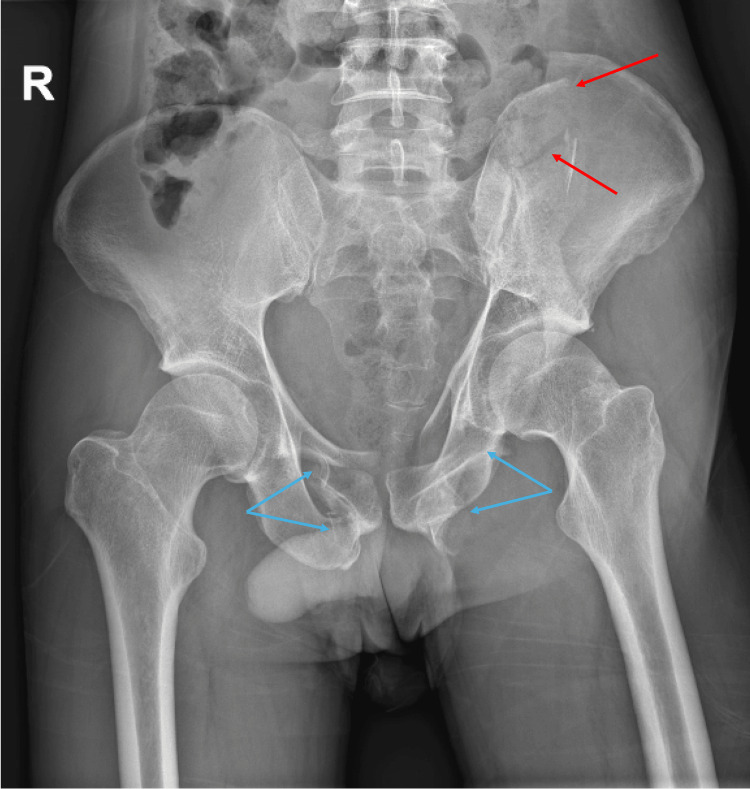
Pre-operative anteroposterior pelvic radiograph Red arrows indicate the left crescent iliac fracture. Blue arrows indicate the bilateral iliopubic and ischiopubic rami fractures.

**Figure 2:**
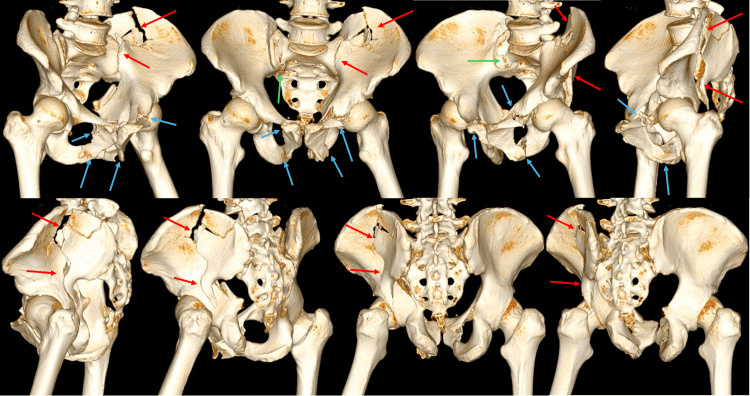
CT of the pelvis with 3D reconstruction shown in multiple views Red arrows indicate the left crescent iliac fracture. Blue arrows indicate the bilateral rami fractures. Green arrows indicate the right sacral ala fracture (Dennis zone I).

**Figure 3:**
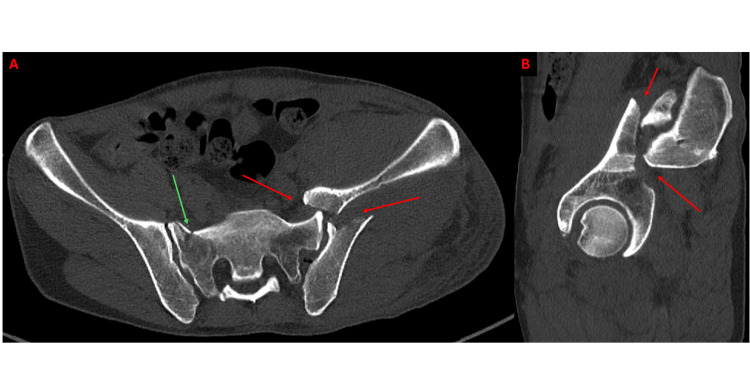
Axial (A) and sagittal (B) CT sections of the crescent-shaped fragment Red arrows indicate the displaced crescent fragment with impaction into the sacral ala. Green arrow (A) indicates the right sacral ala fracture (Dennis zone I).

The patient was admitted to the orthopedic ward for monitoring and preoperative optimization. He remained hemodynamically stable throughout the admission; however, on post-admission day 9, he required transfusion of two units of packed red blood cells (pRBCs) for preoperative hemoglobin optimization, with hemoglobin rising from 10.8 to 11.5 g/dL. Preoperative planning included a detailed review of CT reconstructions and the use of a synthetic sawbone pelvic model (Figure 4), which allowed pre-contouring of the reconstruction plate, simulation of screw trajectories, and anticipation of intraoperative challenges prior to surgery.

**Figure 4:**
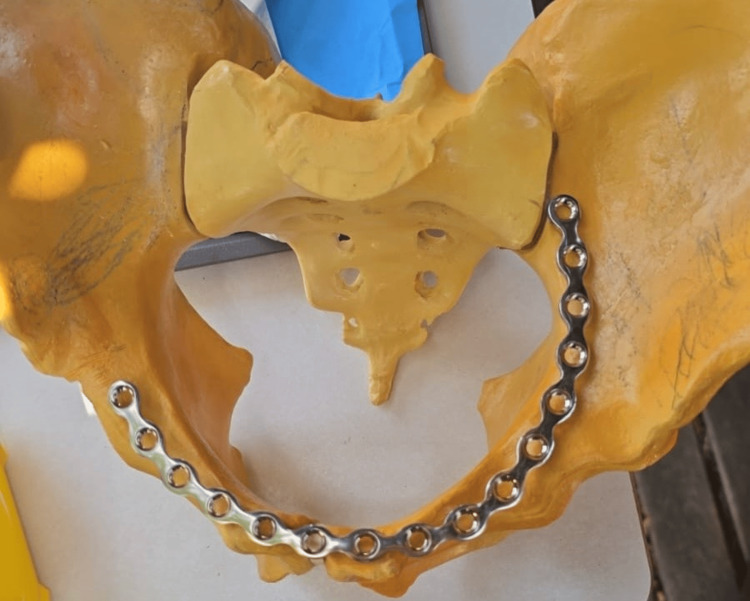
Preoperative planning using a synthetic sawbone pelvic model and a pre-contoured reconstruction plate

Definitive surgical fixation was performed on post-trauma day 10 with the patient in the supine position. A modified Stoppa approach combined with a left iliac window was used to expose the anterior pelvic ring and the left iliac wing fracture. Two Schanz pins were placed as joysticks to assist with rotational control: one in the left iliac crest and one in the left femoral neck. Through the iliac window, a screw was inserted into the left iliac crest to define the height of the mobile iliac fragment while preserving rotational adjustability.

Sequential fixation began with de-impaction of the sacral ala and external rotation of the crescent fragment to achieve reduction. The left iliac wing fracture was then provisionally stabilized with the crest screw. A 16-hole anterior reconstruction plate, pre-contoured on the synthetic sawbone model, was subsequently inserted through the modified Stoppa approach, securing the bilateral rami fractures and the inferior portion of the crescent fragment. An additional screw was placed in the LC-II corridor, running from the anterior inferior iliac spine (AIIS) to the posterior superior iliac spine (PSIS), to provide supplementary rotational stability to the crescent fragment. Posterior ring stabilization was then achieved with a partially threaded left iliosacral screw for SI joint compression, followed by a fully threaded right iliosacral screw for the right sacral ala fracture (Figures 5A-5C). The entire procedure was performed under continuous fluoroscopic control using anteroposterior (AP), inlet, outlet, teardrop, obturator-inlet, down-the-wing, sacral profile, alar, and obturator projections. Total operative time was five hours, with an estimated intraoperative blood loss of 600 mL.

**Figure 5:**
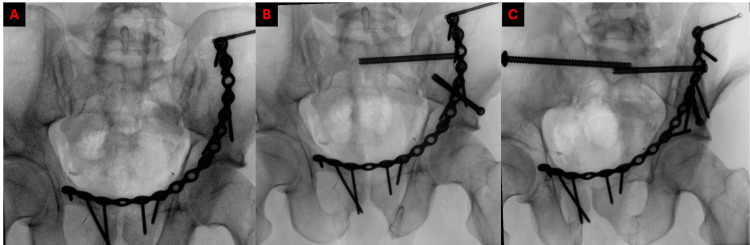
Intra-operative fluoroscopy images of the sequential fixation The images show sequential fixation with an iliac crest screw and 16-hole anterior reconstruction plate (A), followed by the LCII screw and left iliosacral screw (B) and right iliosacral screw (C).

Postoperative radiographs confirmed satisfactory reduction and implant positioning. Rehabilitation followed a structured protocol: non-weight-bearing or tip-toe bearing was maintained during the first two postoperative weeks, followed by progressive weight-bearing as tolerated. By eight weeks, the patient had achieved full weight-bearing without walking aids. He was discharged home on post-operative day 15 with bilateral crutches.

At eight-week follow-up, the patient reported no pain, no functional limitation (Figures 6A-6D), and no sexual dysfunction, and had fully returned to his occupational activity. Postoperative radiographs in weight-bearing confirmed implant stability and maintained reduction (Figures 7A-7D). Functional outcome was assessed using the Majeed Pelvic Score (MPS) [6], on which he achieved 96 out of 100 points, a score consistent with excellent function, with a 4-point reduction solely attributable to his perception of not having fully returned to his pre-injury work performance level.

**Figure 6:**
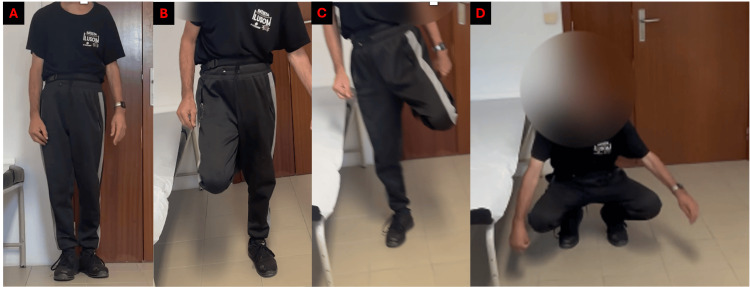
Clinical images of the patients after eight weeks Clinical results at eight weeks demonstrating full ability to stand in two-point (A) and one-point (B and C) stances and squat (D).

**Figure 7:**
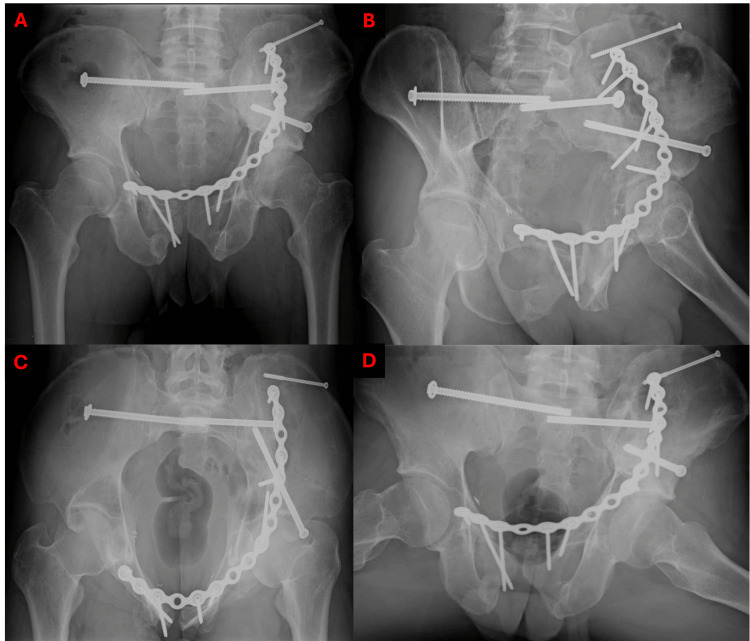
Series of post-operative pelvic X-rays at eight week follow-up (A) Weight-bearing anteroposterior, (B) alar oblique, (C) inlet, and (D) frog-leg lateral views.

## Discussion

Crescent fractures are a diagnostically and technically demanding subset of pelvic ring injuries. Their accurate characterization requires CT with 3D reconstruction, as plain radiographs frequently underestimate the extent of posterior ring disruption [5,7]. In the present case, the combination of a bilateral anterior ring fracture, a left type I crescent fracture with sacral ala impaction, and a right sacral ala fracture within an LCIII pattern required careful preoperative analysis and a staged fixation strategy. The absence of neurological or genitourinary injuries at presentation was reassuring, though their risk must be considered in any LCIII injury given the degree of pelvic instability involved.

The management of complex pelvic ring fractures is best undertaken at dedicated Level I trauma centers by experienced pelvic trauma teams. Such centers provide the multidisciplinary infrastructure, including trauma surgery, orthopedic surgery, interventional radiology, urology, and intensive care, necessary to manage the systemic complications of high-energy pelvic trauma and to perform technically demanding reconstructive procedures safely [1,2]. The outcomes of pelvic fractures have consistently been shown to be superior when treated by surgeons with dedicated expertise in this field, underlining the importance of referral and centralization of care [8].

A defining feature of this case was the use of a synthetic sawbone pelvic model for surgical simulation. This allowed pre-contouring of the reconstruction plate, rehearsal of screw trajectories, and anticipation of intraoperative challenges. In orthopedic trauma, meticulous planning is fundamental to achieving successful outcomes - particularly in pelvic surgery, where anatomical complexity and proximity to neurovascular structures demand precision. In our experience, preoperative model simulation meaningfully reduced intraoperative decision-making time and improved the quality of the reduction achieved. Understanding the underlying mechanism of injury is equally critical: recognition of the impacted crescent fragment and the need for de-impaction prior to fixation were directly informed by biomechanical analysis of the injury.

The modified Stoppa approach provided excellent access to the quadrilateral surface, pelvic brim, and superior pubic rami while minimizing soft-tissue disruption compared to extensile approaches [9]. The addition of a left iliac window allowed direct visualization and reduction of the crescent fragment. A key intraoperative step was the de-impaction of the sacral ala and external rotation of the crescent fragment prior to definitive fixation, a maneuver facilitated by Schanz pins placed in the left iliac crest and femoral neck as joysticks.

The 16-hole anterior reconstruction plate, pre-contoured on the synthetic sawbone model, served a dual purpose, providing definitive fixation of both the anterior ring and the crescent fragment while reducing overall implant burden. An additional screw was placed in the LCII corridor, running from the AIIS to the PSIS, to provide supplementary rotational stability to the crescent fragment [10]. Posterior ring stabilization with iliosacral screws, partially threaded on the left for SI joint compression and fully threaded on the right for sacral ala fixation, is consistent with the established technique for percutaneous posterior pelvic ring fixation [10,11]. Meticulous multiplanar fluoroscopic guidance throughout is indispensable to avoid neurovascular injury during iliosacral screw placement.

The functional outcome at eight weeks was excellent, with a Majeed Pelvic Score of 96/100 [6], well above the threshold of 85 associated with excellent function, and no sexual dysfunction, a complication specifically associated with crescent fractures and one that is frequently underreported in the literature [5]. The structured rehabilitation protocol, which initiated non-weight-bearing or tip-toe bearing in the first two weeks and progressed to full weight-bearing by 8 weeks, was well tolerated and contributed to early recovery. Postoperative weight-bearing radiographs confirmed maintained reduction and implant stability.

## Conclusions

LCIII pelvic ring fractures with a type I crescent fracture-dislocation represent a complex injury with significant risk of functional impairment if suboptimally managed. The excellent outcome achieved in this case reflects the convergence of several critical factors: management at a Level I trauma center by a multidisciplinary team with dedicated pelvic trauma expertise; thorough understanding of the injury mechanism and its implications for surgical planning; meticulous preoperative planning using CT reconstruction and synthetic sawbone model simulation for plate pre-contouring; and a staged, sequential fixation strategy tailored to the specific fracture pattern. The absence of sexual dysfunction at follow-up, a recognized complication of crescent fractures, further underscores the value of anatomical reduction and stable fixation. These factors, in combination with a structured rehabilitation protocol, enabled full functional recovery by eight weeks.
